# Molecular Identification of *Babesia* and *Theileria* Infections in Livestock in the Qinghai–Tibetan Plateau Area, China

**DOI:** 10.3390/ani14030476

**Published:** 2024-02-01

**Authors:** Yihong Ma, Yingna Jian, Geping Wang, Xiuping Li, Guanghua Wang, Yong Hu, Naoaki Yokoyama, Liqing Ma, Xuenan Xuan

**Affiliations:** 1 National Research Center for Protozoan Diseases, Obihiro University of Agriculture and Veterinary Medicine, Inada-cho, Obihiro 080-8555, Japan; 2 Qinghai Academy of Animal Sciences and Veterinary Medicine, Centre for Biomedicine and Infectious Diseases, Qinghai University, Xining 810016, China

**Keywords:** *Theileria* spp., *Babesia* spp., Qinghai–Tibaten plateau area, China

## Abstract

**Simple Summary:**

The Qinghai–Tibetan Plateau (QTPA), in the northwestern region of China, is characterized by diverse geographical features, earning it the title of “the roof of the world”. Despite this, limited information exists on the distribution of tick-borne pathogens in this region. This study aimed to evaluate the infection rates of *Babesia* and *Theileria* species in QTPA. Blood samples collected from livestock species (*n* = 366) were analyzed using different PCR-sequencing techniques. Results showed a high infection rate of *Theileria* spp. (38.2%). *B. motasi*-like Lintan/Ningxia/Tianzhu was detected in 0.3% of samples. Notably, this study reported infection rates of *Babesia* and *Theileria* species in goats, horses, and donkeys in the Qinghai–Tibetan Plateau for the first time.

**Abstract:**

The northwestern region of China, known as the Qinghai–Tibet Plateau Area (QTPA), is characterized by unique climate conditions that support the breeding of various highly-adapted livestock species. Tick vectors play a significant role in transmitting *Babesia* and *Theileria* species, posing serious risks to animal health as well as the economy of animal husbandry in QTPA. A total of 366 blood samples were collected from Tibetan sheep (*n* = 51), goats (*n* = 67), yaks (*n* = 43), cattle (*n* = 49), Bactrian camels (*n* = 50), horses (*n* = 65), and donkeys (*n* = 40). These samples were examined using conventional and nested PCR techniques to detect *Theileria* and *Babesia* species. The overall infection rates were 0.3% (1/366) for *Babesia* spp. and 38.2% (140/366) for *Theileria* spp. Notably, neither *Babesia* nor *Theileria* species were detected in donkeys and yaks. The infection rates of *Babesia* and *Theileria* species among animals in different prefectures were significantly different (*p* < 0.05). Furthermore, *Babesia bovis*, *B. bigemina*, *B. caballi*, and *B. ovis* were not detected in the current study. To our knowledge, this is the first documented detection of *Theileria luwenshuni* infection in Bactrian camels and goats, as well as *T. sinesis* in cattle and *T. equi* in horses on the Qinghai plateau. These novel findings shed light on the distribution of *Babesia* and *Theileria* species among livestock species in QTPA.

## 1. Introduction

The Qinghai–Tibet Plateau area (QTPA), located in the northwestern region of China and with an elevation exceeding 4500 m (14,800 ft) above sea level, is defined by challenging environmental conditions. Renowned for its lofty altitudes and frigid temperatures, it has earned the distinguished titles of “the roof of the world” and “the third pole of the Earth” [1]. The high altitude and thin air in this region give rise to distinct climatic conditions, leading to significant variations in weather patterns. Frequent occurrences of strong winds, minimal yearly temperature fluctuations, and notable daily temperature variations are observed in this area [2].

The QTPA is home to a diverse range of livestock species adapted to the high altitude and cold climate [3], including Tibetan sheep, goats, yaks, cattle, horses, camels, and donkeys [4]. Within the QTPA, the cohabitation of various animal species in shared pastures presents a potential risk for interspecies pathogen transmission, including tick-borne pathogens (TBPs). Additionally, the Qinghai plateau, with a focal point at Qinghai Lake, functions as a vital migratory route, intersecting the Central Asian Flyway from western Siberia through central Asia to India and the East Asian Flyway from Russia through eastern China to Australia [5]. This route is frequented by 292 various bird species, including passerines such as Przevalski’s redstart, slaty-blue flycatcher, great rosefinch, Tarim Babbler, and others, during their respective migration seasons. As passerines can be parasitized by Ixodid ticks, they serve as carriers, contributing to the dissemination of tick-borne pathogens [6,7], which may include infections such as *Babesia* spp. [8]. With complex topography and diverse landscapes, Qinghai features grasslands covering 54.8% of its total area, wetlands at 27.01%, and forests at 4.8% [9], creating an environment conducive to tick proliferation and raising the potential risks of transmitting diverse tick-borne pathogens to livestock [10]. *Babesia* and *Theileria*, two genera of TBPs, have a significant impact on the health of livestock as well as the economy of animal husbandry in the QTPA.

Theileriosis, caused by *Theileria* species, poses significant limitations to the development of livestock husbandry in China. Among cattle, the most prevalent and virulent pathogens associated with bovine theileriosis are *T. sergenti* and *T. annulata*. In sheep and goats, *T. lestoquardi*, *T. luwenshuni*, and *T. uilenbergi* are recognized as highly pathogenic species [11]. Within Qinghai, six species of *Theileria*, including *T. ovis*, *T. sinensis*, *T. luwenshuni*, *T. uilenbergi*, *T. equi*, and *Theileria* sp. *OT3*, have been detected, infecting yaks, Tibetan sheep, and camels [12,13,14,15]. Notably, the detection of *T. sinensis* in yaks from Qinghai was reported for the first time in 2020 [10].

Babesiosis represents a globally pervasive tick-borne disease stemming from the invasion and infection of red blood cells by *Babesia* species [16]. In China, ovine babesiosis is mainly caused by *B. ovis* and *B. motasi*-like Lintan/Ningxia/Tianzhu [17]. The most prevalent species infecting both cattle and water buffaloes are *B. bovis* and *B. bigemina* [18,19]. In Gansu province, yaks have been found to be infected with *B. bigemina* [20] and *B. bovis* [14]. Three *Babesia* species, namely *B. motasi*, *B. bigemina*, and *B. caballi*, were detected in sheep and wild yaks in Qinghai [21,22,23]. 

While prior research in the Qinghai region has delved into the study of *Babesia* and *Theileria*, it predominantly focused on ticks [12,24], yaks [13,14,25], and Tibetan sheep [13,15]. In contrast, this study employs a more comprehensive approach, investigating various key livestock species in the Qinghai region, including Tibetan sheep, goats, yaks, cattle, Bactrian camels, horses, and donkeys. Moreover, the analysis considers the distinct administrative divisions within Qinghai Province. Thus, the primary objective of this study is to evaluate the infection rates of *Babesia* and *Theileria* species affecting livestock in the QTPA.

## 2. Materials and Methods

### 2.1. Sample Collection and Preparation

**Figure 1:**
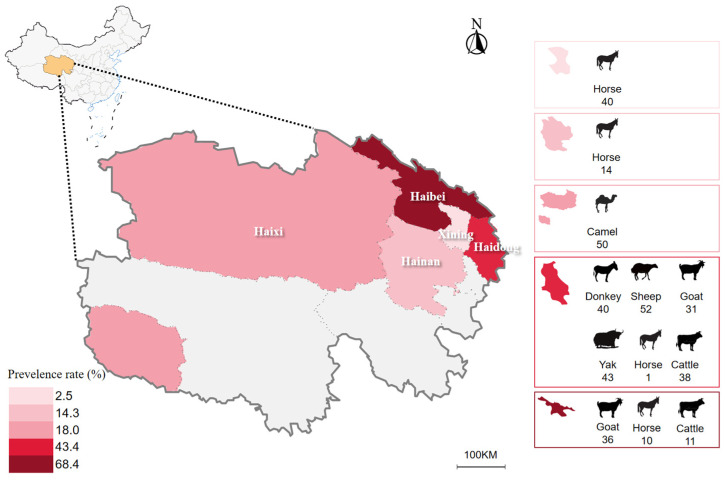
Sample map of Qinghai province and the infection rates across different sampling areas within the Qinghai plateau. Colored regions indicate the areas from which blood samples were collected from livestock animals. The figure was generated and modified using Datav Atlas (https://datav.aliyun.com/portal/school/atlas (accessed on 9 June 2023)).

In this study, a total of 366 blood samples from livestock were randomly collected from five different prefectures in the QTPA between May 2020 and January 2022. The sample size used was estimated based on a representative sample size of 1/200,000 of the total livestock populations on the Qinghai plateau [26]. Additionally, considerations were made based on the prevalent areas for ticks, such as regions abundant in water sources, covered by forests, and used for grazing. The livestock species included in this study were Tibetan sheep (*n* = 51), goats (*n* = 67), yaks (*n* = 43), cattle (*n* = 49), Bactrian camels (*n* = 50), horses (*n* = 65), and donkeys (*n* = 40). The sampling areas encompassed the following regions: (1) Haixi Mongolian and Tibetan Autonomous Prefecture (latitude 35°01′–39°19′ N, longitude 90°07′–99°46′ E), (2) Haibei Tibetan Autonomous Prefecture (latitude 36°44′00″–39°05′18″ N, longitude 98°5′00″–102°41′03″ E), (3) Hainan Tibetan Autonomous Prefecture (latitude 34°38′–37°10′ N, longitude 98°55′–105°50′ E), (4) Haidong (latitude 35°25.9′–37°05′ N, longitude 100°41.5′–103°04′ E), and (5) Xining (latitude 36°13′–37°28′ N, longitude 100°52′–101°54′ E) (Figure 1, Table S1). All protocols were carried out according to the ethical guidelines approved by the Obihiro University of Agriculture and Veterinary Medicine (Permit for animal experiment: 22–23). Livestock owners were invited to take part in this study, and their oral consent was obtained before sample collection.

Genomic DNA was extracted from blood samples using the TIANamp Genomic DNA Kit (Tiangen, Beijing, China) according to the manufacturer’s manual. The DNA concentration was measured using a Biochrom WPA Biowave DNA Life Science Spectrophotometer (Biochrom, Cambrige, UK) and stored at −20 °C until further use.

### 2.2. Molecular Detection of Theileria and Babesia Species

*Theileria* and *Babesia* species were detected using primers listed in Table 1. Briefly, a PCR reaction was prepared with a total volume of 10 μL. This included 1 μL of the DNA template, 0.5 μL of forward and reverse primers (100 μM), 0.2 μL of deoxyribonucleotide triphosphate (200 μM; New England BioLab, Ipswich, MA, USA), 1 μL of 10× ThermoPol Reaction Buffer (New England BioLab, USA), 0.1 μL of Taq polymerase (0.5 U; New England BioLab, USA), and double-distilled water up to 10 μL [15]. The thermal cycling condition for each PCR reaction was carried out following the protocols outlined in previously published studies mentioned in Table 1. PCR products were subsequently subjected to electrophoresis in a 1.5% agarose gel, stained with ethidium bromide, and visualized by UV transillumination.

### 2.3. Sequencing Reaction

For the sequencing protocol, the PCR product of some positive samples was chosen. All positive samples for each pathogen were sequenced if their numbers were fewer than ten. However, if their numbers were more than ten, we sequenced at least 30% of the positive samples from different study areas. These samples represented different livestock species from various sampling areas included in this study. The PCR product of the positive samples was purified using the EasyPure Quick Gel Extraction Kit (TransGen Biotech, Beijing, China) and cloned into *E. coli* DH5α cells (Takara, Shiga, Japan) using the pMD™18-T Vector Cloning Kit (Takara, Shiga, Japan). Plasmid purification was performed using the EasyPure^®^ Plasmid MiniPrep Kit (TransGen Biotech, Beijing, China) according to the manufacturer’s manual. Subsequently, at least three positive clones were sent to Genewiz company, Suzhou, China, for sequencing.

The good-quality sequence reads obtained in this study were compared with published sequences deposited in the GenBank databases (https://blast.ncbi.nlm.nih.gov/Blast.cgi (accessed on 7 October 2022)) using the BLASTn search tool. Phylogenetic trees were constructed using the maximum likelihood method in MEGA X with the bootstrap method, employing 1000 replications [32].

### 2.4. GenBank Accession Numbers

Accession numbers for the sequences generated in this study were obtained through depositing in the GenBank database of the National Center for Biotechnology Information (NCBI). The assigned accession numbers for the sequences generated in this study are listed in Table 2.

### 2.5. Statistical Analyses

The comparison of infection rates for *Babesia* and *Theileria* species detected in this study was conducted using the R 4.3.1 software. Statistical analyses were performed using the multifactorial analysis of variance (ANOVA) test. Statistical significance was determined when the resulting *p*-values were <0.05.

## 3. Results

### 3.1. Overall Infection Rates

The overall infection rates were 0.3% (1/366) for *B. motasi-like* (*Babesia* sp. L/N/T) and 38.2% (140/366) for *Theileria* spp. *B. bovis*, *B. bigemina*, *B. caballi*, and *B. ovis* were not detected in this study.

**Figure 2:**
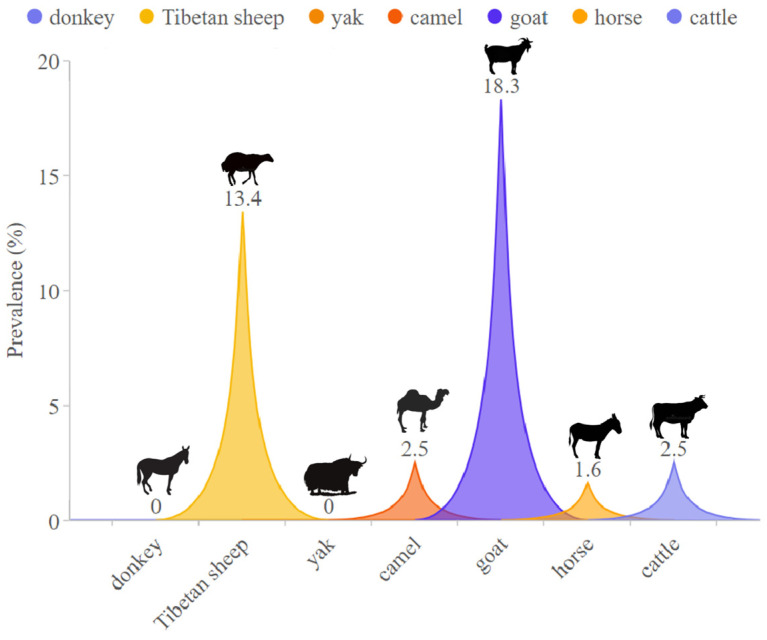
Infection rates of tick-borne protozoa among different animal species examined in the study.

In this study, *Babesia* species (*B. motasi-like*) was detected only in goats. *Theileria* species were detected in 94.2% (49/52) of Tibetan sheep, 100% (67/67) of goats, 9% (6/65) of horses, 18% (9/49) of cattle, and 18% (9/50) of Bactrian camels (Table 3). Neither *Babesia* nor *Theileria* species were detected in donkeys and yaks (Figure 2). Despite this, there was no significant difference in infection rates among different animal species.

The data analysis revealed that Haibei prefecture had the highest infection rate among the examined areas in this study (68.4%; 39/57). In contrast, Xining had the lowest infection rate (2.5%; 1/40) (Figure 1). The infection rates of *Babesia* and *Theileria* from different prefectures show significant differences (*p* = 0.0359) (Table S2).

### 3.2. Sequencing Analyses

In this study, we detected only one *Babesia* species (*Babesia* cf. *motasi*) as well as seven *Theileria* species (including *Theileria* sp. *OT3*, *T. luwenshuni*, *T. uilenbergi*, *T. capreoli*, *Theileria* sp. *Iwate*, *T. sinensis*, and *T. equi*). The BLASTn search revealed that the 18S rRNA sequences of *Theileria* spp. exhibited 99.1–100.0% identities with *T. luwenshuni* (MN394815), 99.5–99.8% with *T. uilenbergi* (MN394818), 100.0% with *Theileria* sp. *OT3* (MG930118), 100% with *T. sinensis* (MN628025), and 99.7–99.8% with *T. equi* (MT093500). Furthermore, one *Theileria* spp. isolate from sheep showed 98.6% homology with *T. capreoli* from China (KJ188219), and an isolate from goat displayed 97.98% homology with *Theileria* sp. *Iwate* from Japan (AB602881). The *rap-1b* sequence of *Babesia* cf. *motasi* exhibited 99.7% identity with *Babesia* cf. *motasi* detected in sheep from Qinghai province, China (MH908949) (Table 2).

### 3.3. Phylogenetic Analyses

**Figure 3:**
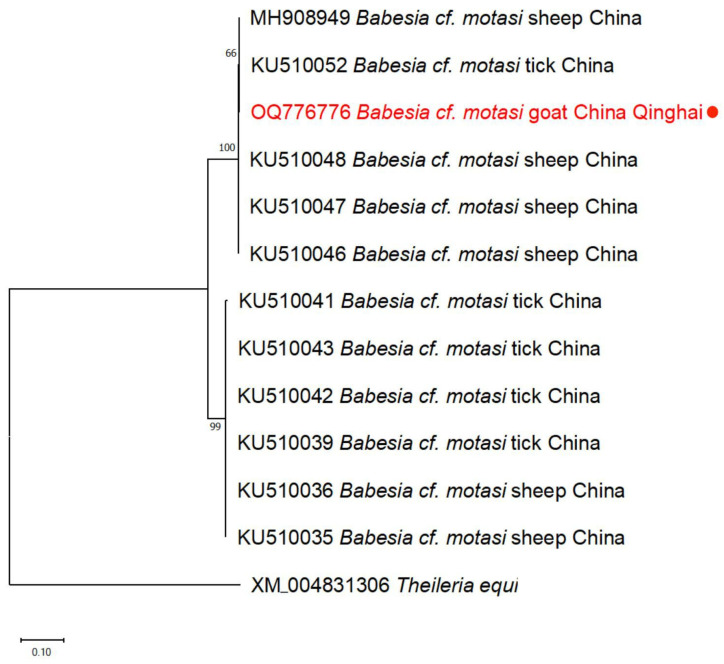
Phylogenetic tree of *Babesia* cf. *motasi* based on *rap-1b* sequence analysis. The tree was constructed with Maximum likelihood method using MEGA X with Tamura 3-parameter model, and numbers at nodes represent percentage of occurrence of clades in 1000 bootstrap replications of data. The sequences obtained in this study and their accession numbers are marked in red with dot superscript. *T. equi* (XM004831306) was used as an outgroup.

The phylogenetic analyses of the *Babesia* cf. *motasi* sequence (OQ776776) based on the *rap-1b* gene revealed its placement in the same clade as *Babesia* cf. *motasi* isolates from sheep and tick samples in China (Figure 3).

**Figure 4:**
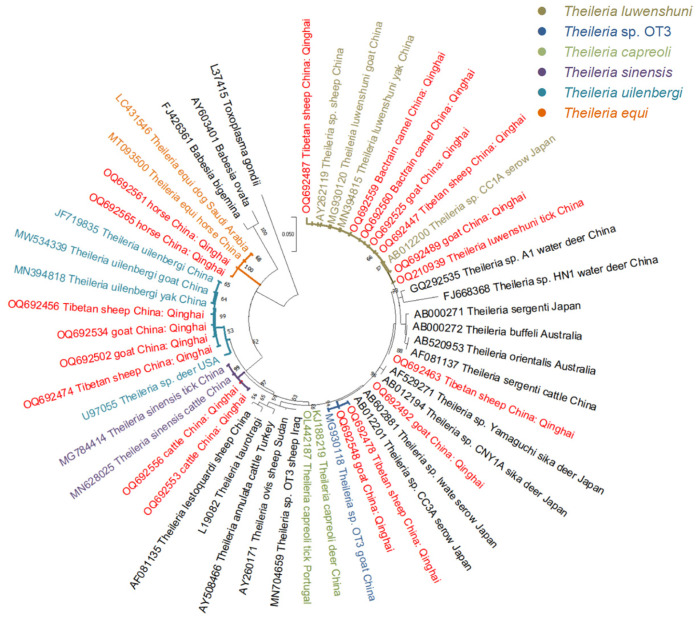
Phylogenetic tree of *Theileria* species based on 18S rRNA sequence analysis. The tree was constructed with Maximum likelihood method using MEGA X with Tamura 3-parameter model, and numbers at nodes represent percentage of occurrence of clades in 1000 bootstrap replications of data. Different colors represent different *Theileria* species, as shown in figure legend. The sequences obtained in this study and their accession numbers are marked in red with dot superscript. *B. microti* (AB190459) was used as an outgroup.

Additionally, our study yielded significant findings regarding the 18S rRNA sequences of *Theileria* species. We classified *Theileria* species isolates from sheep and goats as *T. uilenbergi*, *T. luwenshuni*, and *Theileria* sp. *OT3*, based on their placement within the same clade. On the other hand, all cattle isolates were placed with *T. sinensis* isolates from cattle and tick populations in the neighboring Gansu province [19]. Notably, this is the first detection of *T. sinensis* in Qinghai, a region located near the QTPA. Furthermore, the phylogenetic analyses of horse isolates confirmed their infection with *T. equi*, supporting existing knowledge in this regard. Importantly, our research also revealed the detection of *T. luwenshuni* among Bactrian camels within the QTP area, marking the initial documentation of such an occurrence (Figure 4).

## 4. Discussion

This study sheds light on the prevalence and diversity of *Babesia* and *Theileria* species among various livestock species in the challenging environment of the QTPA. The QTPA, renowned for its extreme altitude and harsh climate, hosts a diverse range of livestock species, contributing to the complexity of interspecies interactions and potential transmission of TBPs. In the Qinghai Plateau, a total of 31 tick species belonging to 6 genera and 2 families have been documented. Among them, *Dermacentor nuttalli* and *Haemaphysalis qinghaiensis* emerge as the predominant tick species in Qinghai Province, exhibiting extensive distribution across most regions [33], which potentially facilitates *Babesia* [24] and *Theileria* [12,24] transmission on the Qinghai plateau.

Previous studies have reported the presence of different *Babesia* species in the Qinghai Plateau, including *B. motasi*, *B. bigemina*, and *B. caballi*, infecting sheep, yaks, and ticks [15,25]. In the current study, only one of the examined goats was positive for *Babesia motasi-like Lintan/Ningxia/Tianzhu*, giving the possibility that the Qinghai plateau area has a low infection rate with *Babesia* species. This finding contributes to our understanding of the distribution of these pathogens in the region. Earlier investigations detected six different species of *Theileria*, namely *T. ovis*, *T. sinensis*, *T. luwenshuni*, *T. uilenbergi*, *T. equi*, and *Theileria* sp. *OT3*, in the Qinghai Plateau area [12,13,14,15]. In this study, we identified a wide range of *Theileria* species with an infection rate of 38.2% (140/366). Through sequencing, we identified various *Theileria* species, including *Theileria* sp. *OT3*, *T. luwenshuni*, *T. uilenbergi*, *T. capreoli*, *Theileria* sp. *Iwate*, *T. sinensis*, and *T. equi*. The results highlight that *T. luwenshuni* exhibits the highest infection rate among the different *Theileria* species. This suggests that *T. luwenshuni* may be the predominant species responsible for infecting livestock in Qinghai Plateau domestic animals. Notably, we present the first documented evidence of *T. sinensis* presence in cattle from the Qinghai Plateau. A previous study has shown its detection in yaks in Qinghai [14]. Furthermore, our analysis provides confirmation of *T. equi* infection in horses within the Qinghai plateau, marking the initial recorded identification of *T. equi* in horses within this region.

Our comprehensive approach, spanning across Tibetan sheep, goats, yaks, cattle, Bactrian camels, horses, and donkeys, provides a more inclusive understanding compared to previous research primarily focused on specific animals. Furthermore, we consider distinct administrative divisions within Qinghai Province, adding granularity to our analysis. Goats and Tibetan sheep were found to be more susceptible to infection with *Theileria* spp., possibly due to variations in susceptibility and immune responses [34], whereas TBPs were not detected in yaks and donkeys. Yaks demonstrate a reduced prevalence or potential resistance to *Theileria* spp., a phenomenon likely attributed to specific immune mechanisms [35], notably high-altitude-induced modifications in blood composition. The heightened levels of erythrocytes and hemoglobin [36], may influence *Theileria* (erythrocyte parasite) infection. Additionally, the dense and long hair of yaks acts as a physical barrier, hindering tick attachment and reducing the likelihood of *Theileria* spp. transmission. The statistical analysis of the multifactorial analysis of variance (ANOVA) does not show statistical significance for the observed differences in infection rates among different livestock in the region (*p* = 0.063 > 0.05). Although the majority of animals exhibit good health, we have identified the presence of *Babesia* and *Theileria* in their blood samples. This observation may imply potential infection risks or indicate varying susceptibility levels among certain individuals to these parasites. Consequently, the transmission dynamics of these parasites in the animal population will be further investigated, and potential prevention and control strategies will be explored. Additionally, the findings of this study revealed that Haibei Prefecture, renowned for hosting the crucial avian migration route of Qinghai Lake [5] and boasting abundant water resources [37], exhibited the highest prevalence of *Theileria* among the scrutinized regions. Rigorous statistical analyses further underscored a statistically significant difference in infection rates across the diverse study areas (*p* < 0.05). To gain a deeper understanding of the potential factors influencing the infection rate of TBPs in QTPA, future investigations could consider expanding the sample size, implementing more extensive data collection methods, or exploring alternative statistical approaches.

In summary, this study focused on the molecular detection of *Theileria* and *Babesia* species in various livestock on the Qinghai–Tibetan Plateau. The findings underscore the importance of monitoring tick-borne pathogens in this region. However, it is essential to acknowledge certain limitations in our study. Firstly, the blood samples from donkeys were collected from various farmers within the same region who practiced intensive management instead of extensive management, resulting in reduced tick exposure compared to grazing. Additionally, the inclusion of donkey blood from different farms within the same region introduces constraints on its representativeness [38]. Secondly, while most animals appeared healthy, our study’s design hinders the identification of potential infection risk factors. Asymptomatic carriers may be present, and the lack of visible signs of illness does not guarantee the absence of exposure or infection, impacting our understanding of the epidemiological landscape. Thirdly, potential diagnostic biases exist, as variations in methods or sensitivity may influence tick-borne pathogen detection rates. This introduces uncertainty in our results, emphasizing the need for careful interpretation due to the complexities of diagnostic procedures. Future studies should explore factors influencing *Babesia* and *Theileria* infection rates in the QTPA by incorporating broader sample sets and diverse diagnostic methods. This approach will contribute to the refinement of prevention and control strategies in this challenging environment.

## 5. Conclusions

This study significantly contributes to our understanding of the prevalence and diversity of *Babesia* and *Theileria* species within the challenging environment of the Qinghai–Tibet Plateau Area (QTPA). By investigating a broad spectrum of livestock species, including Tibetan sheep, goats, yaks, cattle, Bactrian camels, horses, and donkeys, we offer a more comprehensive perspective compared to previous research focused on specific animals. Noteworthy findings include the low infection rate of *Babesia* species in the Qinghai Plateau area, with only one examined goat testing positive for *Babesia motasi-like Lintan/Ningxia/Tianzhu*. *Theileria* species exhibited a higher infection rate of 38.2%, with *T. luwenshuni* identified as the predominant species. Our study also presents the first documented evidence of *T. sinensis* in cattle and *T. equi* in horses within the Qinghai Plateau. Notably, distinct susceptibility patterns were observed among livestock species, with goats and Tibetan sheep being more susceptible to *Theileria* spp., while yaks and donkeys showed resistance. The observed prevalence of *Babesia* and *Theileria* in apparently healthy animals suggests varying susceptibility levels among individuals, prompting further investigation into transmission dynamics and the exploration of potential prevention and control strategies. Additionally, the regional analysis revealed that Haibei Prefecture exhibited the highest prevalence of *Theileria*, emphasizing the need for more in-depth studies to understand the factors influencing infection rates across diverse regions in the QTPA.

## Figures and Tables

**Table 1:** The list of primer sequences used in this study.

Pathogen	Target Gene	Method	Primer Sequence (5′→3′)		Annealing Temperature (°C)	Amplicon Size (bp)	Reference
*B. bovis*	*sbp*-4	PCR	F	AGTTGTTGGAGGAGGCTAAT	55	907	[27]
			R	TCCTTCTCGGCGTCCTTTTC			
		nPCR	nF	GAAATCCCTGTTCCAGAG	55	503	
			nR	TCGTTGATAACACTGCAA			
*B. bigemina*	*rap*-1a	PCR	F	GAGTCTGCCAAATCCTTAC	55	879	[27]
			R	TCCTCTACAGCTGCTTCG			
		nPCR	nF	AGCTTGCTTTCACAACTCGCC	55	412	
			nR	TTGGTGCTTTGACCGACGACAT			
*B. caballi*	*bc48*	PCR	F	GGCTCCCAGCGACTCTGTGG	63	570	[28]
			R	CTTAAGTGCCCTCTTGATGC			
*B. motasi-like* *(Babesia* sp. *L/N/T)*	*rap*-1b	PCR	F	TGCGCCTTCGAGTTGTACAAGAG	58	765	[29]
			R	GACGGGTTGCRTAGGCTGAC			
		nPCR	nF	TGCGTGGAAGATAGAAAGTTAGCC	60	536	
			nR	ATGACTGATCTCGACTCTCCATTAGCTGG			
*B. ovis*	ssu rRNA	PCR	F	TGGGCAGGACCTTGGTTCTTCT	62	549	[30]
			R	CCGCGTAGCGCCGGCTAAATA			
*Theileria* spp.	18S rRNA	PCR	F	GAAACGGCTACCACATCT	55	778	[31]
			R	AGTTTCCCCGTGTTGAGT			
		nPCR	nF	TTAAACCTCTTCCAGAGT		581	
			nR	TCAGCCTTGCGACCATAC			

**Table 2:** GenBank accession numbers were assigned to *Theileria* and *Babesia* species sequences generated in this study.

Obtained Sequences					The Closest BLASTn Match		
Pathogen	Isolation Source	Target Gene	GenBank Number	Length (bp)	Identity (%)	Pathogen	GenBank Number (Host, Country)
*Theileria* spp.	Sheep	18s rRNA	OQ692447	584	99.14%	*T. luwenshuni*	MN394815 (yak, China)
	Sheep	18s rRNA	OQ692473	586	98.64%	*T. capreoli*	KJ188219 (deer, China)
	Sheep	18s rRNA	OQ692474	580	99.66%	*T. uilenbergi*	MN394818 (yak, China)
	Sheep	18s rRNA	OQ692478	589	100%	*Theileria* sp. *OT3*	MG930118 (goat, China)
	Goat	18s rRNA	OQ692489	582	99.83%	*T. luwenshuni*	MN394815 (yak, China)
	Goat	18s rRNA	OQ692492	593	97.98%	*Theileria* sp. *Iwate*	AB602881 (serow, Japan)
	Goat	18s rRNA	OQ692502	579	99.66%	*T. uilenbergi*	MN394818 (yak, China)
	Goat	18s rRNA	OQ692548	589	100%	*Theileria* sp. *OT3*	MG930118 (goat, China)
	Cattle	18s rRNA	OQ692553	579	100%	*T. sinensis*	MN628025 (cattle, China)
	Horse	18s rRNA	OQ692565	583	99.83%	*T. equi*	MT093500 (horse, China)
	Camel	18s rRNA	OQ692560	582	100%	*T. luwenshuni*	MN394815 (yak, China)
*Babesia* spp.	Goat	*rap-1b*	OQ776776	535	99.63%	*Babesia* cf. *motasi*	MH908949 (sheep, China)

**Table 3:** The detection rates of *Theileria* and *Babesia* species among livestock in QTPA.

Area	Animal Species	TBP ^1^	*Babesia* spp.					*Theileria* spp.
		*n*	*B.* *bigemina*	*B.* *bovis*	*B.* *caballi*	*Babesia* cf. *motasi*	*B.* *ovis*	
Haidong	Donkey	40	-	-	-	-	-	-
	Tibetan sheep	52	-	-	-	-	-	49 (94.2)
	Goat	31	-	-	-	1 (3.2)	-	31 (100.0)
	Yak	43	-	-	-	-	-	-
	Horse	1	-	-	-	-	-	-
	Cattle	38	-	-	-	-	-	9 (23.7)
	Subtotal	205	-	-	-	1 (0.5)	-	89 (43.4)
Haibei	Goat	36	-	-	-	-	-	36 (100.0)
	Horse	10	-	-	-	-	-	3 (30.0)
	Cattle	11	-	-	-	-	-	-
	Subtotal	57	-	-	-	-	-	39 (68.4)
Hainan	Horse	14	-	-	-	-	-	2 (14.3)
	Subtotal	14	-	-	-	-	-	2 (14.3)
Haixi	Bactrian camels	50	-	-	-	-	-	9 (18.0)
	Subtotal	50	-	-	-	-	-	9 (18.0)
Xining	Horse	40	-	-	-	-	-	1 (2.5)
	Subtotal	40	-	-	-	-	-	1 (2.5)
Total		366	1 (0.3)					140 (38.2)

^1^ The data are represented in terms of the number of infected individuals (infection rate (%)).

## Data Availability

Data are contained within the article.

## Supplementary Materials

The following supporting information can be downloaded at: https://www.mdpi.com/article/10.3390/ani14030476/s1, Table S1: The blood sample collected in different places from Qinghai Plateau; Table S2: The effect of livestock species and localities on infection rate of TBPs.
