# Supplementary material for: Molecular Identification of Babesia and Theileria Infections in Livestock in the Qinghai–Tibetan Plateau Area, China
Source: Animals (Basel). 2024 Feb 1;14(3):476. doi: 10.3390/ani14030476 (PMC10854629; doi:10.3390/ani14030476)
Supplement: Supplementary file 1 [file animals-14-00476-s001.zip › animals-2802525-supplementary.pdf]

Table S1. The blood sample collected in different places from Qinghai Plateau.

State	County	Site	Longitude	Latitude	Animals	Sample size	Date
Haidong	Huzhu	1	102.4607	36.79175	yak	10	2021.11.23
					goat	21	2021.11.23
					cattle	11	2021.11.23
		2	102.526	36.90187	Tibetan sheep	9	2021.03.24
		3	102.1807	36.71019	yak	30	2021.11.20
		4	102.0253	36.67823	donkey	40	2021.11.20
		5	102.5543	36.90635	cattle	25	2022.01.17
	Ledu	6	102.6691	36.56436	yak	3	2021.04.13
					horse	1	2021.04.13
		7	102.4585	36.35392	goat	10	2021.03.25
		8	102.4176	36.55348	Tibetan sheep	10	2021.03.25
		9	102.6423	36.52647	Tibetan sheep	1	2021.04.13
		10	102.6294	36.50118	Tibetan sheep	2	2021.04.13
	Minhe	11	102.7278	36.34168	Tibetan sheep	17	2021.03.23<br>2021.04.15
					cattle	2	2021.04.15
		12	102.6449	36.23601	Tibetan sheep	13	2021.04.08
Haixi	Wulan	13	97.86457	36.65661	Bactrian camel	29	2020.05.20
		14	98.89838	36.84857	Bactrian camel	21	2020.05.20
Hainan	Gonghe	15	100.811	36.63234	horse	14	2021.11.26
Haibei	Menyuan	16	101.9287	37.22693	goat	36	2021.12.07
		17	101.857	37.3184	cattle	11	2021.12.07
	Gangcha	18	100.2144	37.29197	horse	10	2021.12.01
Xining	Huangzhong	19	101.3547	36.56464	horse	10	2021.11.25
	Datong	20	101.7264	36.91613	horse	30	2021.12.03

Table S2. The effect of livestock species and localities on infection rate of TBPs.

	**Area**			**Livestock**		
	**Area**	**Pathogens**	**Residuals**	**Livestock**	**Pathogen**	**Residuals**
**Df**[1]	4	11	44	7	11	77
**Sum Sq**[2]	481.4	1241.8	1873.0	375.1	948.0	2039.9
**Mean Sq**[3]	120.4	112.9	42.6	53.6	86.2	26.5
**F value**[4]	2.827	2.652		2.023	3.253	
**Pr (>F)**[5]	0.036	0.011		0.063	0.001	

[1] Df: degrees of freedom; 2 Sum Sq: sum of squares; 3 Mean Sq: represents mean squares; 4 F value: F-statistic; 5 Pr(>F): p-value associated with the F-statistic.
